# Characterising the Mucosal and Systemic Immune Responses to Experimental Human Hookworm Infection

**DOI:** 10.1371/journal.ppat.1002520

**Published:** 2012-02-09

**Authors:** Soraya Gaze, Henry J. McSorley, James Daveson, Di Jones, Jeffrey M. Bethony, Luciana M. Oliveira, Richard Speare, James S. McCarthy, Christian R. Engwerda, John Croese, Alex Loukas

**Affiliations:** 1 Queensland Tropical Health Alliance and School of Public Health and Tropical Medicine, James Cook University, Cairns, Queensland, Australia; 2 Princess Alexandra Hospital, Brisbane, Queensland, Australia; 3 George Washington University, Washington D.C., United States of America; 4 Anton Breinl Centre, James Cook University, Townsville, Queensland, Australia; 5 Queensland Institute of Medical Research, Brisbane, Queensland, Australia; 6 The Townsville Hospital and James Cook University, Townsville, Queensland, Australia; University of Manchester, United Kingdom

## Abstract

The mucosal cytokine response of healthy humans to parasitic helminths has never been reported. We investigated the systemic and mucosal cytokine responses to hookworm infection in experimentally infected, previously hookworm naive individuals from non-endemic areas. We collected both peripheral blood and duodenal biopsies to assess the systemic immune response, as well as the response at the site of adult worm establishment. Our results show that experimental hookworm infection leads to a strong systemic and mucosal Th2 (IL-4, IL-5, IL-9 and IL-13) and regulatory (IL-10 and TGF-β) response, with some evidence of a Th1 (IFN-γ and IL-2) response. Despite upregulation after patency of both *IL-15* and *ALDH1A2*, a known Th17-inducing combination in inflammatory diseases, we saw no evidence of a Th17 (IL-17) response. Moreover, we observed strong suppression of mucosal *IL-23* and upregulation of *IL-22* during established hookworm infection, suggesting a potential mechanism by which Th17 responses are suppressed, and highlighting the potential that hookworms and their secreted proteins offer as therapeutics for human inflammatory diseases.

## Author Summary

Parasitic worms reside in the gastrointestinal tracts of billions of humans in developing countries. Despite the enormous disease burdens associated with these infections, very little is known about the immune response in the gut tissue of humans to these parasites. We conducted a clinical trial where we obtained gut biopsies from people experimentally infected with hookworms and present here the first report of the immune response by healthy human gut tissue to a parasitic worm. We show that hookworms suppress the production of pro-inflammatory molecules and promote the expression of anti-inflammatory and wound healing molecules in the gut, providing a potential mechanism by which parasitic worms reside for long periods in their human hosts and suppress inflammation associated with auto-immune diseases.

## Introduction

The hookworms *Necator americanus* and *Ancylostoma duodenale* infect an estimated 740 million people, mostly in tropical regions of the world, causing significant burden of disease [1]. As for most Neglected Tropical Diseases (NTDs), there is currently no prophylactic or therapeutic vaccine against hookworm infection, although clinical trials are underway on a number of promising candidate antigens [2]. Despite efforts to eliminate this disease from developing countries, experimentally-induced hookworm infection offers potential as an anti-inflammatory therapy for human autoimmune [3] and allergic [4], [5] diseases. However, despite their importance in regards to disease burden in resource poor countries (especially in children and women of child bearing years) and their potential as an anti-inflammatory therapy for use in industrialized countries, little is known about the mucosal immune responses of humans to hookworm, or indeed any other gastrointestinal (g.i.) helminths parasites.

Unlike many other human g.i. helminths, despite a robust, parasite-specific immune response, naturally acquired protection against hookworm is only partially effective at best; indeed, in endemic areas the oldest people often have the heaviest worm burdens [6], [7]. Nonetheless, previous studies on people naturally infected with hookworm have identified associations between reduced egg counts and Th2 responses. For example, IL-5 production correlates positively with resistance to reinfection after anthelmintic drug cure [8], and levels of IgE reactive against defined larval antigens are negatively associated with hookworm egg counts [9].

A small number of experimental infections in hookworm naive, healthy human volunteers have been conducted, with an exclusive focus on the systemic immune response at both the humoral and cellular levels [10]–[12], and gross observations of the gut via capsule endoscopy [3]. These earlier observations described the onset of eosinophilia, production of parasite specific IgG and IgE, and secretion of both Th1 (IFN-γ and TNF-α) and Th2 (IL-5 and IL-13) cytokines. With the onset of patency, IL-10 was produced and T cell proliferation was blunted and was not restored until long after curative therapy [7], [11].

Most of our understanding of mucosal immunity to g.i. nematodes comes from studies in laboratory mice. Th2 cytokines are required for resistance to many g.i. helminths, as seen in mice that are genetically deficient in Th2 cytokines and associated signalling molecules [13], [14]. In the draining lymph node, Th2 cytokines are responsible for class-switching of B cells to IgG1 and IgE, as well as recruiting and activating innate immune cells and blocking parasite effector molecules [15]. At the site of adult worm residence in mice, the duodenum, Th2 cytokines are responsible for increased mucus and fluid production in the gut and smooth muscle contractility, which increases ejection of parasites [16], [17]. They also lead to recruitment, expansion and differentiation of innate immune cells such as eosinophils, alternatively activated macrophages, mast cells and basophils in the gut which can directly or indirectly lead to ejection of parasites [15]. Thus differentiation of Th2 cells and production of Th2 cytokines, both systemically and in the mucosa, may be important for intestinal parasite clearance in mice.

Th1 (and Th17) responses are also induced during some helminth parasite infections. In the absence of a Th2 response, or where Th1/Th17 responses have been artificially upregulated, an uncontrolled Th1/Th17 response to schistosomes leads to acute pathology and ultimately death in mice [18], [19]. Thus, it has been proposed that the Th2 response generated during schistosomiasis may downregulate Th1/Th17 responses, leading to suppression of immunopathology and survival of the host [15]. Suppression of Th17 responses by Th2 cytokines in the mucosa has also been shown in mice infected with g.i. nematodes [20], prompting the suggestion that nematodes may ameliorate inflammatory gut diseases by dampening pro-inflammatory Th17 responses.

We previously reported a study using human hookworm infection to treat celiac disease [21]. Although no overt suppression of clinical pathology was detected, suppression of gluten-specific inflammatory Th1 and Th17 responses was seen in the mucosa [22]. After established hookworm infection but prior to challenge with gluten, samples were taken from control and hookworm infected individuals, and here we prospectively collected data on the hookworm-specific cytokine responses in the peripheral circulation and, for the first time, the duodenal mucosa, of hookworm naive individuals before and after controlled experimental infection with *N. americanus*. This is the first description of the mucosal immune response of humans to hookworms; indeed, other than a case study where an individual patient with active ulcerative colitis was treated with whipworm and the mucosal immune response was assessed [23], this is the first report of the mucosal immune response in healthy volunteers in a clinical trial to experimental infection with helminths, and provides valuable information to support the development of both vaccines against hookworm infection and hookworm-derived peptidic therapies for inflammatory diseases.

## Methods and Materials

### Ethics statement

The Princess Alexandra Hospital, Queensland Institute of Medical Research and Townsville Hospital Human Research Ethics Committees approved the study. Written informed consent was obtained from all subjects.

### Clinical protocol

The methods used for our placebo-controlled, blinded clinical trial using hookworm to treat celiac disease have been described elsewhere [21]. Briefly, twenty confirmed HLA-DQ2+ celiac disease sufferers on a long-term gluten-free diet (and therefore in remission for celiac disease) were recruited, randomised into 2 groups and either infected with 10 infective larvae (L3) of *N. americanus* (“hookworm” group) or given a placebo of topical chilli (Tabasco sauce) (“control” group). Twelve weeks later, a booster infection of 5 infective larvae (or a placebo infection) was administered. At week 20 post-prime infection, all individuals were given a gluten challenge consisting of four slices of white bread per day for 5 days. This trial will herein be referred to as “Trial 1”. Approximately 6 months after the end of Trial 1 (during which all participants returned to a strict gluten-free diet), seven of the ten control subjects (those who did not receive hookworm in Trial 1) participated in a continuation trial: two could not participate due to other commitments, and one could not participate due to raised tissue transglutaminase antibodies. These 7 participants were infected with *N. americanus*, boosted and challenged with gluten in an identical manner to that described for Trial 1. This trial will herein be referred to as “Trial 2”.

**Figure 1:**
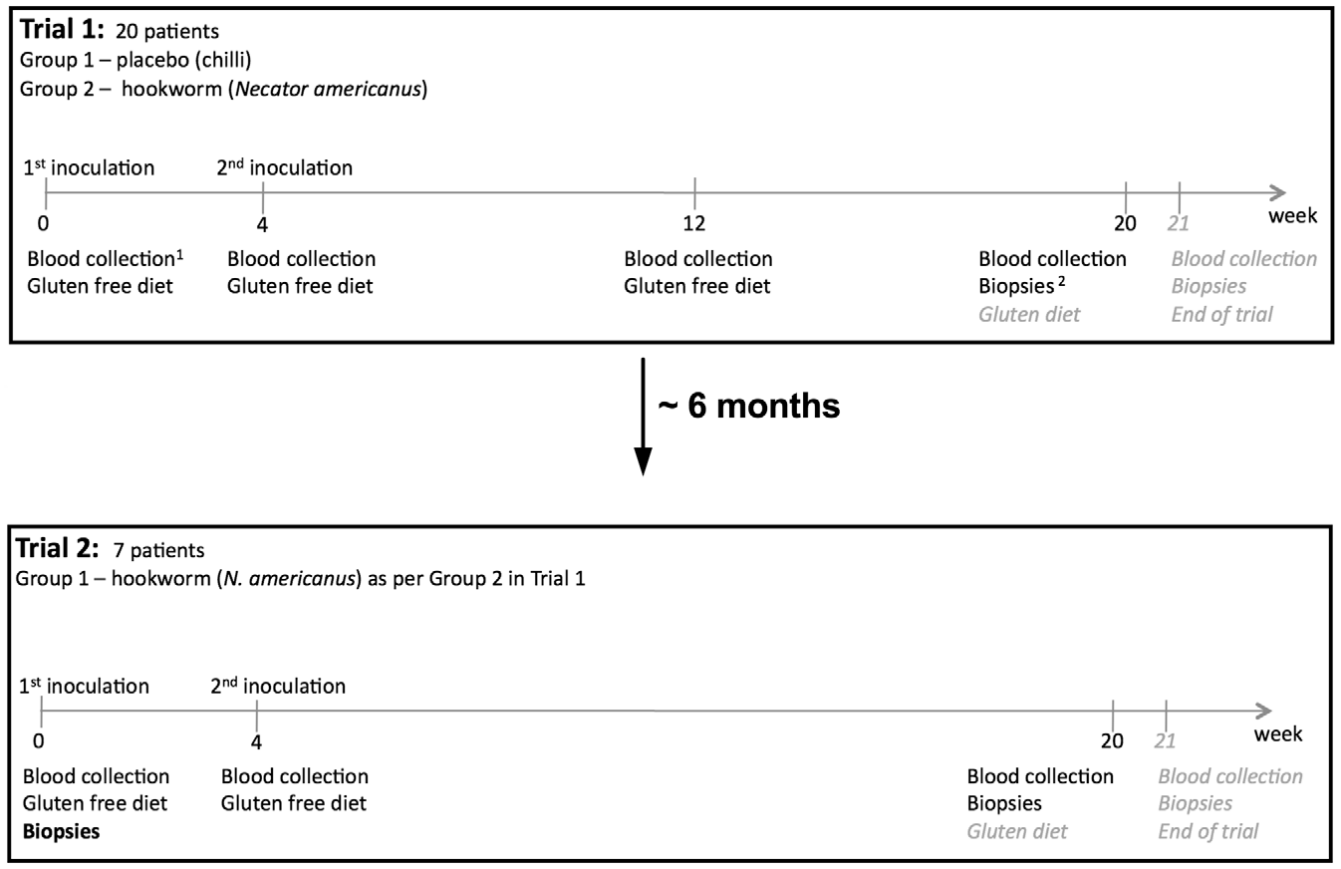
Trial design. For Trial 1, 20 patients were chosen according to selection criteria [21] and divided into two groups of 10, placebo control and hookworm infected (HW). All 20 patients completed the trial. For Trial 2, seven of the 10 patients from Group 1 in Trial 1 (placebo) chose to participate in the next trial and received hookworm infection and gluten challenge in an identical manner to that described for Group 2 in Trial 1. The only difference was that gut biopsies were taken at week 0 (prior to hookworm infection; denoted in bold font) in addition to biopsies taken at weeks 20 and 21. For the purposes of this study, sample collection ceased at week 20 (prior to gluten administration) and tissues from week 21 were not utilized, except for samples taken from the worm attachment site which were collected at week 21; week 21 tissues were critical for assessment of the effect of hookworm on the anti-gluten response in celiac disease and are therefore highlighted in italicised grey font and have been reported in relevant publications [21], [22]. ^1^Forty millilitres of blood per person were collected at weeks 0, 4, 12 (Trial 1 only), 20 and 21. ^2^Gut biopsies were taken at weeks 0 (Trial 2 only), 20 (pre-gluten ingestion) and 21 (post-gluten ingestion) using a gastroscope as described elsewhere [21].

In both trials hookworm infection was confirmed in all subjects by either fecal egg counts and/or identification of adult parasites in the duodenum during endoscopy [21]. The structure of the trials is summarised in Figure 1.

### Peripheral blood mononuclear cell acquisition and antigen restimulation

Peripheral blood mononuclear cells (PBMCs) were isolated from blood drawn into heparinised tubes over a Ficoll-Paque Plus gradient (GE Healthcare) as described in Figure 1. Cells were cultured for 120 h at 37°C, 95% O_2_/5% CO_2_ at 2.5×10^5^ cells/well in round-bottom 96-well plates in Tissue Culture Medium (Med: RPMI 1640, 10% fetal bovine serum, 100 U/µl penicillin, 100 µg/ml streptomycin and 2 mM L-glutamine), in the absence (“Med”) or presence of 10 µg/ml *N. americanus* excretory/secretory proteins (NaES). NaES was prepared as previously described [24], and was depleted of endotoxin using two rounds of phase separation using Triton-X114 [25]. Endotoxin levels in NaES after depletion were assessed using E-toxate (Sigma); levels were below the detection limit of the assay (<0.05–0.1 EU/ml) for stock solution of NaES at a concentration of 1.77 mg/ml. Cell-free supernatants were collected and analysed using a Cytometric Bead Array (CBA; BD Biosciences). Antigen-specific production of cytokines was determined by subtraction of baseline cytokine levels from unstimulated PBMCs (Med) from those stimulated with NaES.

### Biopsy culture

Duodenal biopsies were taken from week 20 post-prime infection (prior to gluten challenge) in both groups from Trial 1, and also from sites adjacent to (within 0.5 cm) a hookworm attachment site where adult hookworms were found in the upper duodenum (5 of 10 hookworm-infected individuals) by endoscopy [21]. In Trial 2, biopsies were taken at week 0 (prior to infection) and at week 20 post-prime infection. Whole biopsies were placed in wells of a 24-well plate containing 500 µl MED alone or MED containing 10 µg/ml NaES, and cultured for 24 h in 95% O_2_/5% CO_2_ at 37°C. Cell-free supernatants were taken and analysed using a Cytometric Bead Array (BD Biosciences). Biopsies were then placed into Trizol (Invitrogen) and RNA was purified following the manufacturer's protocols.

### Quantitative real-time RT-PCR

For quantitative real-time RT-PCR (qPCR), RNA was prepared from biopsies in Trial 2 by the phenol-chloroform method (Trizol). mRNA quality was tested using a Bioanalyzer (Agilent) or agarose gel electrophoresis prior the reverse-transcription step. cDNA was prepared using Superscript III reverse transcriptase (Invitrogen) according to the manufacturer's protocol. PBMCs from a healthy donor were cultured for 24 h in 95% O_2_/5% CO_2_ at 37°C with phytohemagglutinin-A (PHA) and used to create standard curves and positive controls. Levels of transcripts were normalised to the housekeeping gene β-actin and are presented as arbitrary units. SyBr Green mastermix (Qiagen) was used in a Rotor-Gene Q thermal cycler (Qiagen) according the manufacturer's protocol. Primers used for each gene product are listed in Table S1.

### Statistical analyses

All analyses were carried out using Prism 5.0 (Graphpad). Paired data were compared by Wilcoxon matched-pairs signed rank test; 3 or more sets of paired data were compared by the Kruskal-Wallis non-parametric ANOVA. Unless otherwise indicated, differences were not significantly different. N.S. = Not Significant, * = p<0.05, ** = p<0.01, *** = p<0.001. All error bars show the standard error of the mean.

## Results

### Th2 immune responses to hookworm infection

**Figure 2:**
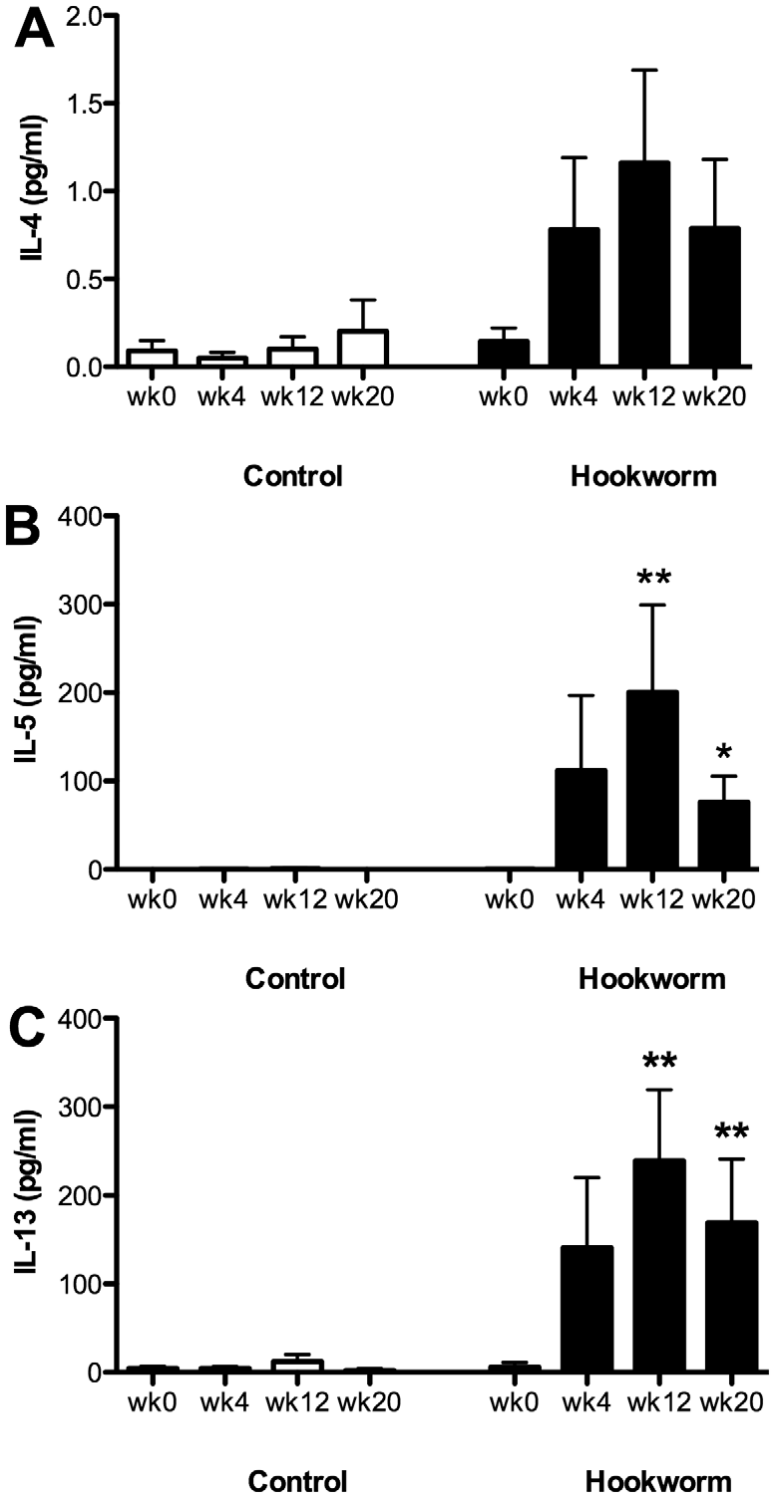
Systemic production of hookworm-specific cytokines. Peripheral blood mononuclear cells were harvested from subjects in Trial 1 and cultured for 120 h at 37°C in either tissue culture medium (MED) or MED containing 10 µg/ml *Necator americanus* ES products (NaES). Cell supernatants were removed and levels of IL-4 (A), IL-5 (B) and IL-13 (C) determined using a Cytometric Bead Array. Cytokine levels from PBMCs stimulated with just MED alone were subtracted from those stimulated with NaES. Data were analysed by Kruskal-Wallis non-parametric ANOVA, comparing time points at weeks 4, 12 and 20 to week 0 within each group.

All volunteers infected with hookworm were confirmed to have active infections using a combination of capsule endoscopy (to visualize adult worms in the gut) and/or the presence of eggs in the feces [21]. PBMCs from volunteers infected with 15 third-stage larvae (L3) of *N. americanus* were restimulated with NaES and showed increased antigen-specific production of the Th2 cytokines IL-4, IL-5 and IL-13, compared with PBMCs from uninfected controls, reaching a peak 12 weeks after infection (Figure 2A–C), although increases in IL-4 levels did not reach statistical significance. These data indicate that, as expected, hookworm infection induces a systemic Th2 response.

**Figure 3:**
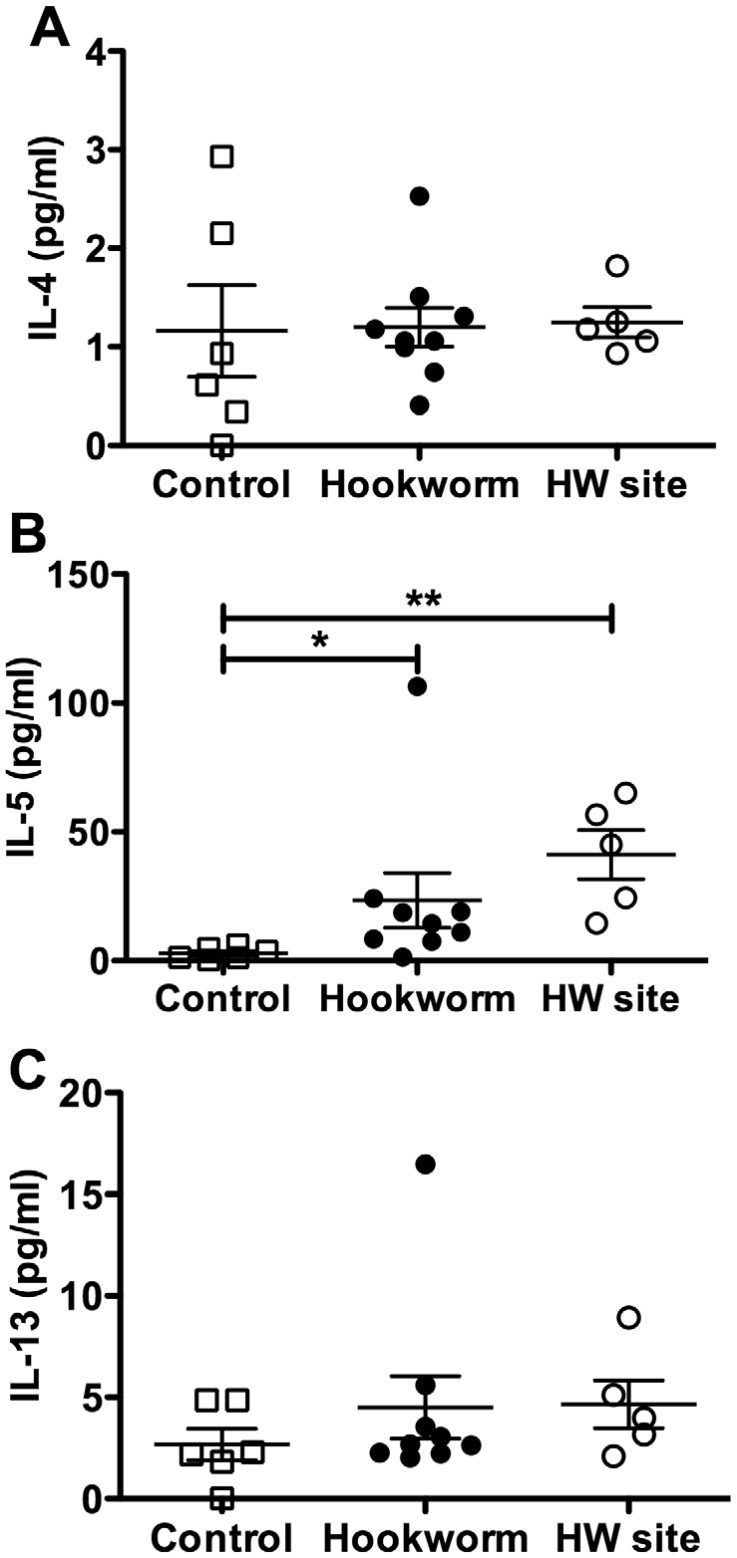
Production of Th2 cytokines in the duodenal mucosa of hookworm infected individuals. Duodenal biopsies from Trial 1, taken from either the duodenum at week 20 post-infection or from directly adjacent to an adult hookworm attachment site (HW site – determined by endoscopy) at week 21 in the hookworm group only, were cultured for 24 h in tissue culture medium at 37°C with 95% O_2_/5% CO_2_. Cell supernatants were removed and levels of IL-4 (A), IL-5 (B) and IL-13 (C) were determined using a Cytometric Bead Array. Data were analysed by Mann-Whitney U test.

To establish whether this Th2 response was present at the site of adult worm residence, duodenal biopsies were taken at week 20 after hookworm infection (immediately prior to gluten challenge). Biopsies were also taken from directly adjacent to the hookworm attachment site at week 21 (after gluten challenge) from 5 of the 10 hookworm-infected individuals where adult worms were observed by endoscopy. All biopsies were cultured without stimulation and supernatants were removed for cytokine analysis. Biopsies from both control and hookworm infected individuals produced similar levels of IL-4 and IL-13 (Figure 3A and C). However, significantly increased levels of IL-5 were produced by biopsy cells from hookworm-infected individuals, especially those biopsies taken adjacent to the hookworm attachment site (Figure 3B). The increased levels of IL-5 at the hookworm attachment sites were not the result of gluten challenge, because at week 21, biopsies from sites distal to the hookworm attachment sites produced decreased levels of IL-5 (12.77 pg/ml +/− 13.90) compared to biopsies from week 20 (23.47 pg/ml +/− 31.88) or the hookworm attachment site (41.18 pg/ml +/− 21.33).

### Characterisation of the mucosal immune response before and after hookworm infection

**Figure 4:**
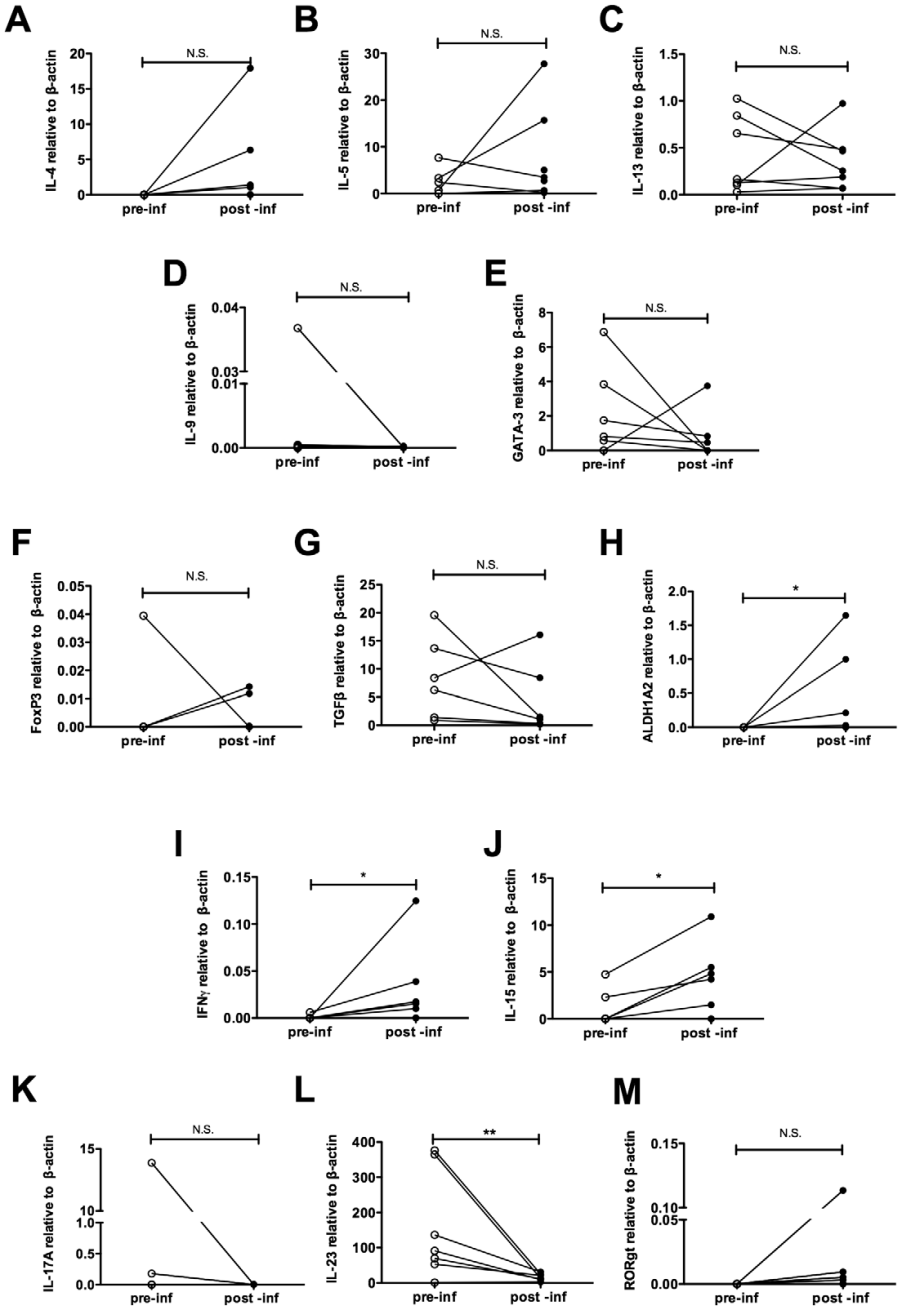
*Ex vivo* cytokine gene expression in the duodenal mucosa of hookworm infected individuals. Duodenal biopsies from Trial 2 were taken before (week 0) and 20 weeks after hookworm infection, and RNA was prepared *ex vivo*. Levels of *IL-4* (A) *IL-5* (B), *IL-13* (C), *IL-9* (D), *GATA-3* (E), *Foxp3* (F), *TGF-β* (G), *ALDH1A2* (H), *IFN-γ* (I), *IL-15* (J), *IL-17A* (K), *IL-23* (L) and *RORγt* (M) transcripts were determined by quantitative real time RT-PCR.

Figures 2 and 3 show that experimental hookworm infection induces a systemic, *Necator* antigen-specific, Th2 response, and a weak but detectable basal mucosal Th2 response. In order to further characterise this response, duodenal biopsies were taken before (week 0) and after an established hookworm infection (week 20 post-prime infection) from infected individuals in Trial 2. Cytokines were measured in the supernatants of duodenal biopsies cultured for 24 h in medium only. There was no significant difference in the protein levels of IL-2, IFN-γ, TNF-α, IL-17A, IL-4, IL-5, IL-10 and IL-13 when comparing wk 0 (pre-infection) to wk 20 (post-infection) (Fig. S1). RNA transcripts were obtained to assess gene expression levels in the absence of *ex vivo* stimulation for a range of cytokines and transcription factors associated with different T helper cell phenotypes. Levels of mRNA encoded by the Th2/Th9 genes *IL-4*, *IL-5*, *IL-13*, *IL-9* and *GATA-3* appeared unaffected by hookworm infection using this technique (Figure 4A–E). Accumulation of mRNA transcribed by the regulatory T cell associated gene *Foxp3* (Figure 4F), or the gene encoding the immunosuppressive cytokine *TGF-β* (Figure 4G), were also unaffected, although levels of *Foxp3* mRNA were below the detection limits of the assay in the majority of the samples tested. However, accumulation of mRNA encoded by the *ALDH1A2* gene was significantly increased after hookworm infection (Figure 4H). *ALDH1A2* encodes retinaldehyde dehydrogenase, an enzyme that is important for production of retinoic acid from vitamin A metabolites. Transcription of the Th1 cytokine gene *IFN-γ* (Figure 4I) and the T cell proliferative cytokine gene *IL-15* (Figure 4J) were also upregulated after hookworm infection. Levels of the Th17-associated genes, *IL-17A* and *RORγt*, were both extremely low, close to or below the detection limit of the assay, but nevertheless appeared unchanged after hookworm infection (Figure 4K and M). Accumulation of mRNA transcribed by the Th17 inducing and stabilising cytokine IL-23, however, was strongly down-regulated after hookworm infection (9.6-fold decrease in the mean value) (Figure 4L).

### Hookworm antigen-specific systemic and mucosal responses

**Figure 5:**
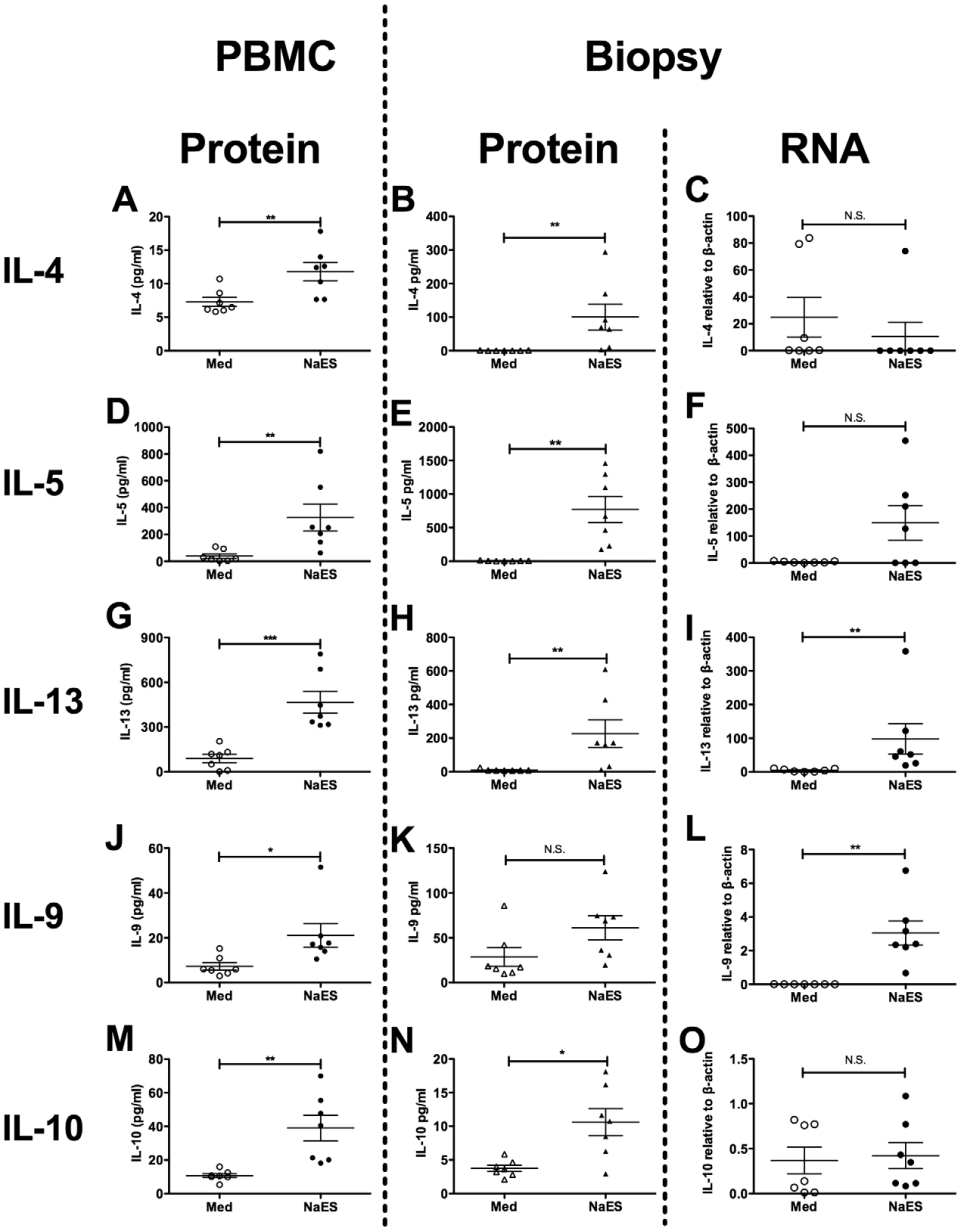
Systemic and mucosal hookworm specific immune responses. Peripheral blood mononuclear cells (PBMCs) from Trial 2 were cultured for 120 h with either tissue culture medium (Med) or Med containing 10 µg/ml *Necator americanus* ES products (NaES). Duodenal biopsies were also taken at week 20 post-infection, and cultured for 24 h at 37°C, 95% O2/5% CO_2_, in either Med alone or 10 µg/ml NaES in Med. Cell-free supernatants were taken from both PBMCs and biopsy cultures and levels of soluble cytokines were determined by cytometric bead array. RNA was also prepared from biopsies after stimulation and quantitative real time RT-PCR was used to determine levels of cytokine gene transcripts. Soluble cytokines released from PBMCs are shown in panels A, D, G, J and M. Biopsy-derived soluble cytokines are shown in panels B, E, H, K and N. Biopsy cytokine transcripts are shown in panels C, F, I, L and O. Cytokine levels determined were IL-4 (A–C), IL-5 (D–F), IL-13 (G–I), IL-9 (J–L) and IL-10 (M–O).

In Trial 1, PBMCs that were restimulated with NaES from hookworm-infected individuals but not uninfected controls produced Th2 cytokines (Figure 2). PBMCs and duodenal biopsies were cultured with NaES or MED alone from all individuals in Trial 2 before and after hookworm infection. Supernatants from both cultures were taken for soluble cytokine analysis, and restimulated biopsies were taken after culture for RNA preparation and qPCR. In pre-infection biopsies (wk0), there was no significant difference in IL-2, IFN-γ, TNF-α, IL-17A, IL-4, IL-5, IL-10 and IL-13 produced after restimulation in culture with NaES compared to medium only (Figure S2). When PBMCs from hookworm-infected participants were restimulated with NaES they produced IL-4, IL-5 and IL-13 (Figure 5A, D, G), as previously shown (Figures 2A–C). We then extended these studies to show that restimulated biopsies also produced these cytokines, both at the levels of secreted protein (Figure 5B, E and H) and RNA transcripts (Figure 5C, F and I), although we did not detect a change for IL-4 transcript levels. PBMCs and biopsies from infected individuals also produced IL-9 and IL-10 in response to NaES (Figure 5J–O), however increased IL-10 production to NaES was not detectable by qPCR.

**Figure 6:**
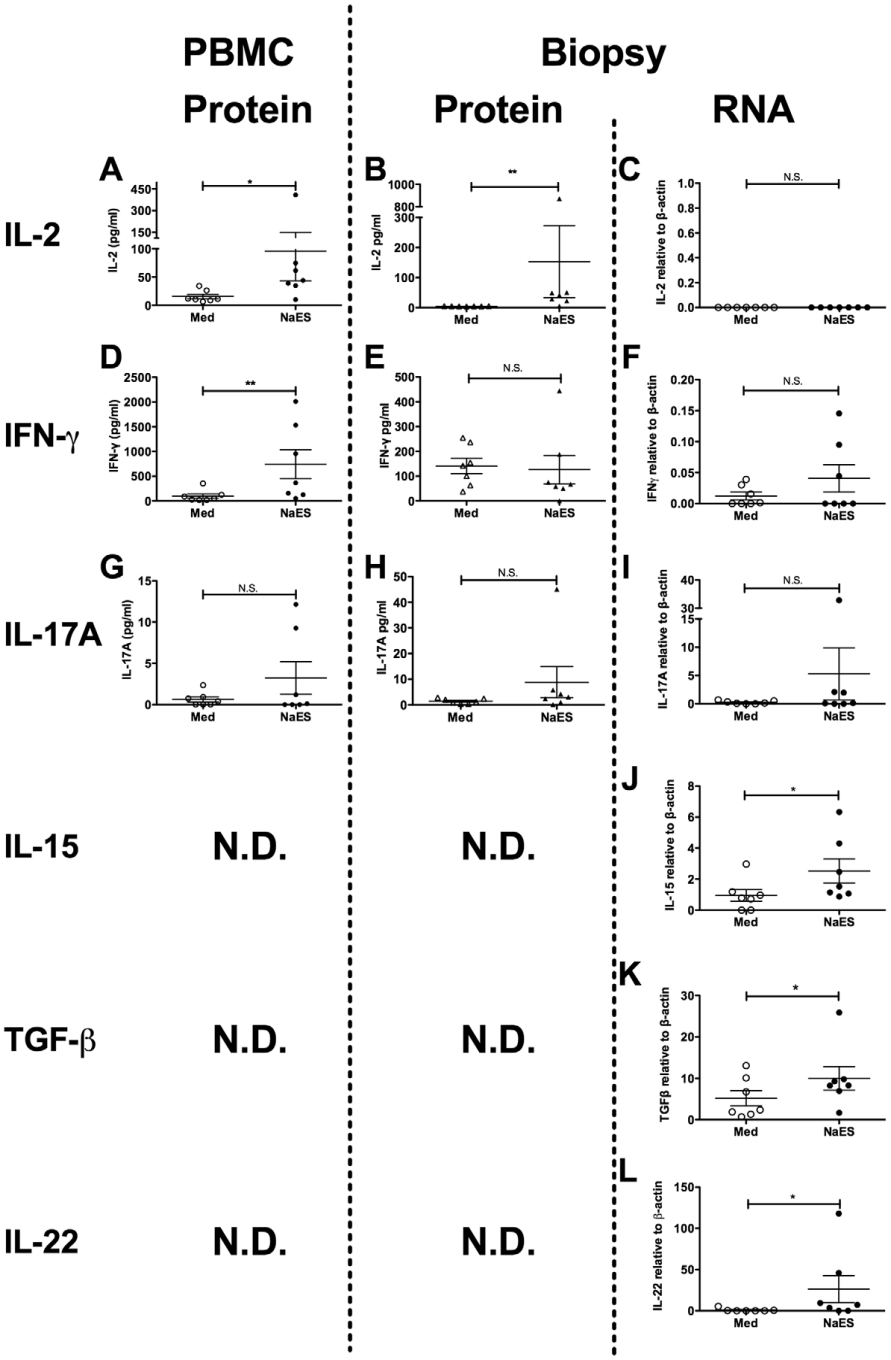
Systemic and mucosal hookworm specific immune responses. Peripheral blood mononuclear cells (PBMCs) from Trial 2 were cultured for 120 h with either tissue culture medium (Med) or Med containing 10 µg/ml *Necator americanus* ES products (NaES). Duodenal biopsies were also taken at week 20 post-infection, and cultured for 24 h at 37°C, 95% O2/5% CO2, in either Med alone or 10 µg/ml NaES in Med. Cell-free supernatants were taken from both PBMCs and biopsy cultures and levels of soluble cytokines determined by Cytometric Bead Array. RNA was also prepared from biopsies after stimulation and quantitative real time RT-PCR was used to determine levels of cytokine gene transcripts. Soluble cytokines released from PBMCs are shown in panels A, D and G. Biopsy-derived soluble cytokines are shown in panels B, E and H. Biopsy cytokine transcripts are shown in panels C, F, I, J and K. Cytokine levels determined were IL-2 (A–C), IFN-γ (D–F), IL-17A (G–I), IL-15 (J), TGF-β (K) and IL-22 (L).

The qPCR data from unstimulated biopsies taken before and after hookworm infection indicated that infection may induce a Th1 response, while suppressing a Th17 response (Figure 4). We also assessed the levels of inflammatory cytokines produced by PBMCs and biopsy cultures when stimulated with NaES. As shown in Figure 6, restimulation with NaES induced upregulation of the proliferative cytokine IL-2 in both PBMCs and biopsy cultures at the protein level (Figure 6A and B), but corresponding levels of mRNA were too low to detect by qPCR (Figure 6C). Restimulation with NaES also induced production of the Th1 cytokine IFN-γ from PBMCs (Figure 6D), but not from biopsy cultures, either at the protein (Figure 6E) or RNA (Figure 6F) levels. We did not detect upregulation of the Th17 cytokine IL-17A from cultures of PBMCs or biopsies restimulated with NaES (Figure 6G–I). The accumulation of mRNA transcribed by another T cell proliferative cytokine gene, *IL-15* (Figure 6J), the immunosuppressive cytokine gene *TGF-β* (Figure 6K) and the wound healing cytokine *IL-22* (Figure 6L), were also increased in NaES restimulated biopsies. Again, biopsies taken prior to hookworm infection did not produce upregulated expression of any of these cytokines upon NaES stimulation (Figure S2).

## Discussion

Here, we present the first description of the mucosal cytokine response of healthy humans to either an experimental or naturally acquired helminth infection. As all individuals in this trial were infected when in established remission of their celiac disease whilst maintaining a strict gluten-free diet, and all analyses presented in this study were performed with blood and tissue collected prior to gluten challenge (excepting data acquired from the adult hookworm attachment site, Figure 3), we regard the subjects as being representative of normal, healthy individuals, and treat our results accordingly.

The polarisation of the T cell response in hookworm infection is of some debate, with some studies showing a mixed Th1/Th2 response, while others report only a polarised Th2 response [12], [26]. These conflicting results might be explained by differences in methods used to assess cytokine levels and antigen preparation [26]. We found a robust Th2 response produced to hookworm antigen as expected, with some evidence of a systemic, but not mucosal hookworm-specific Th1 response. Using qPCR with unstimulated biopsies taken before and after hookworm infection, we showed upregulation of *IFN-γ* transcripts. However, restimulation of post-infection biopsies with NaES did not result in increased *IFN-γ* production. Thus, although an innate IFN-γ response develops after hookworm infection, we could not identify antigen-specific memory Th1 cells in the duodenal mucosa.

We did not detect a significant alteration of levels of mRNAs encoded by the Th17 cytokine *IL-17A* or transcription factor *RORγt* in PBMCs or biopsies. This is surprising, as we observed increased levels of *ALDH1A2* and *IL-15* mRNAs in unstimulated mucosa with further enhancement of *IL-15* mRNA accumulation following stimulation with NaES. Although retinaldehyde dehydrogenase (encoded by *ALDH1A2*) produces the normally immunosuppressive retinoic acid, which imprints gut homing on T cells [27] and switches the pro-inflammatory Th17 to a regulatory response [28], in active celiac disease the impact appears quite the opposite whereby elevated retinoic acid and IL-15 promotes Th1/17 responses in the gut mucosa [29]. We did not detect an increase in Treg markers in response to hookworm infection in this study, or when previously measured by FACS and immunohistochemistry [22], despite a wealth of evidence of Treg induction following experimental helminth infections and administration of ES proteins in mouse models [30]–[32], and naturally acquired infections of humans with other helminths [33], [34].

In active celiac disease, IL-15 is considered a crucial cytokine in maintaining autoimmune (Th1/Th17) pathology, a relationship now recognized somewhat incongruously as being dependent on retinoic acid [29]. In isolation, our IL-15 and *ALDH1A2* data would argue against the potential for hookworm infection to protect against gluten toxicity in celiac disease, the primary incentive for undertaking this clinical trial [21], [22]. However, in contrast to the increased accumulation of IL-2, *IL-15* and *ALDH1A2*, accumulation of mRNA encoding the innate Th17-inducing and stabilising IL-23 was potently suppressed by hookworm infection, potentially neutralising the impact of these Th17 promoting cytokines. Consistent with suppression of *IL-23* mRNA accumulation, Th17 inflammation did not occur. IL-23 is produced by antigen presenting cells under the influence of microbial signals, and is a key cytokine in driving intestinal inflammation [35]. Moreover, IL-23 was recently shown to induce production of pro-inflammatory cytokines by innate lymphoid cells in the gut of patients with Crohn's disease [36]. Thus we hypothesise that hookworm infection suppresses pro-inflammatory cytokine production (such as IL-23) by innate cells in the gut, similarly to that seen in *H. polygyrus* infection in mice where a suppressive dendritic cell subset is expanded in the mucosa [37], and ES proteins from the parasite suppress activation of these cells and subsequent cytokine production [38].

IL-22, an IL-23 dependent Th17 cytokine, acts via the IL-22R expressed on intestinal epithelial cells, promoting innate immunity against bacteria, cell regeneration and tissue healing. In inflammatory bowel disease, high levels of IL-22 are present in inflamed tissue [39]. Interestingly, Broadhurst *et al.* described a case study of a patient with ulcerative colitis who deliberately ingested thousands of eggs of the whipworm *Trichuris trichuria* in which infection ameliorated disease activity, and this effect correlated with increased expression of Th2 cytokines and IL-22 [23]. In our study, biopsies from patients infected with a small number of *N. americanus* larvae showed upregulation of *IL-22* mRNA levels after restimulation with NaES *in vitro*. It is beyond the scope of this discussion to attempt to define what cytokine milieu determines when and what Th17 complex drives inflammation or regulation. It does seem, however, that helminth-stimulated IL-22, perhaps derived from a non-Th17 source, such as NK cells or CD11c+ cells, contributes to the biological relationship between parasite and host, whilst conditioning and promoting a less inflammatory phenotype [23], [40], [41].

Both the regulatory cytokines, TGF-β and IL-10, were induced by hookworm infection, but this was only evident in mucosa restimulated with NaES. During *H. polygyrus* infection in mice, Th2 responses are induced in lamina propria T cells, and Th1 responses in these T cells are inhibited by parasite-induced TGF-β- and IL-10-producing T cells [42]. We may have identified a similar regulatory process, possibly adapted to further fine tune Th2 associated damage at the hookworm attachment site. If the rate of progression and the severity of damage to the mucosa accompanying the worm's attachment is central to determining which population of parasites a particular host will sustain, as has been suggested in an earlier endoscopic study of *N. americanus* survival in experimentally infected humans [3], these inflammation-modifying cytokines almost certainly have a role.

Herein we characterised the systemic and mucosal immune responses to an anthropophilic hookworm infection. As expected, we detected a systemic and mucosal hookworm-specific Th2 response in experimentally infected people. Our data indicate that although an antigen-specific Th1 response was detectable in the blood, no antigen-specific IFN-γ was detectable in the mucosa. Therefore the increased IFN-γ we detected in the mucosa most likely comes from an innate source, possibly NK cells [24]. Despite enhanced production of IL-15 and *ALDH1A2*, levels of *IL-23* were dramatically suppressed after hookworm infection, possibly accounting for the absence of a Th17 response via suppression of antigen presenting cell function.

## Supporting Information

Figure S1 **Cytokine production in the duodenal mucosa of hookworm infected individuals.** Duodenal biopsies from Trial 2 were taken pre-infection (week 0) and 20 weeks after hookworm infection (post-infection). Protein in the supernatant was measured 24 h after incubation at 37°C 5%CO_2_ with medium only. Levels of IL-2 (A) IFN-γ (B),TNF-α (C), IL-17A (D), IL-4 (E), IL-5 (F), IL-10 (G) and IL-13 (H) were determined by cytokine bead array (BD Biosciences). No significant differences were seen before and after infection.
(TIFF)

Figure S2 **Cytokine production prior to hookworm infection in the duodenal mucosa after restimulation with NaES.** Duodenal biopsies from Trial 2 were taken before (week 0) hookworm infection. Cytokines in the supernatant were measured 24 h after incubation at 37°C 5%CO_2_. Levels of IL-2 (A) IFN-γ (B), TNF-α (C), IL-17A (D), IL-4 (E), IL-5 (F), IL-10 (G)and IL-13 (H) were determined by Cytokine Bead Array (BD Biosciences). No significant differences were seen cytokine levels produced in cultures restimulated with NaES.
(TIFF)

Table S1 **Sequences of primers used for real-time RT-PCR.**
(DOC)
