# Supplementary figures and images for: Characterising the Mucosal and Systemic Immune Responses to Experimental Human Hookworm Infection
Source: PLoS Pathog. 2012 Feb 9;8(2):e1002520. doi: 10.1371/journal.ppat.1002520 (PMC3276555; doi:10.1371/journal.ppat.1002520)

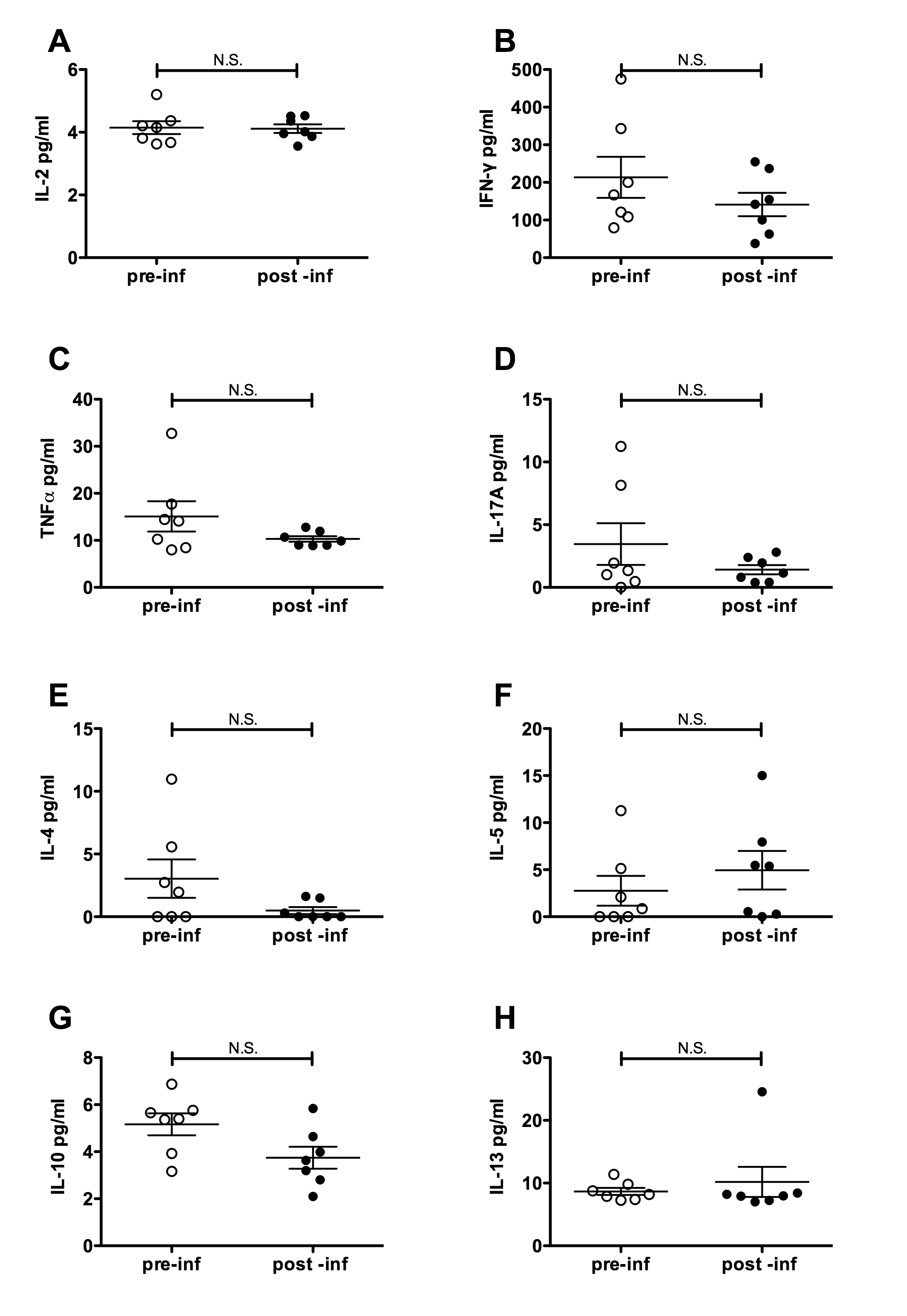

Supplement: Figure S1 — Cytokine production in the duodenal mucosa of hookworm infected individuals. Duodenal biopsies from Trial 2 were taken pre-infection (week 0) and 20 weeks after hookworm infection (post-infection). Protein in the supernatant was measured 24 h after incubation at 37°C 5%CO2 with medium only. Levels of IL-2 (A) IFN-γ (B),TNF-α (C), IL-17A (D), IL-4 (E), IL-5 (F), IL-10 (G) and IL-13 (H) were determined by cytokine bead array (BD Biosciences). No significant differences were seen before and after infection. (TIFF) [file ppat.1002520.s001.tif]

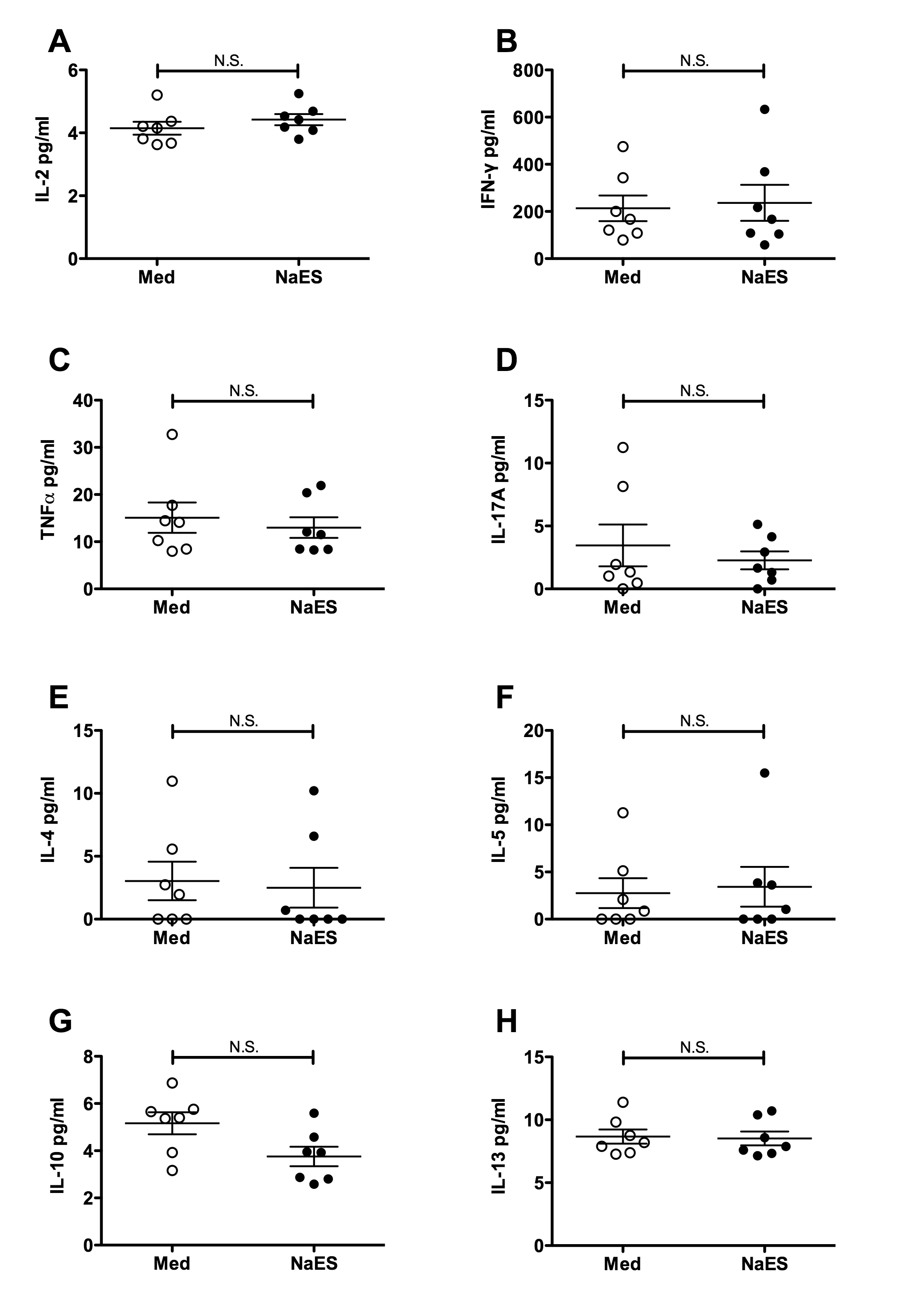

Supplement: Figure S2 — Cytokine production prior to hookworm infection in the duodenal mucosa after restimulation with NaES. Duodenal biopsies from Trial 2 were taken before (week 0) hookworm infection. Cytokines in the supernatant were measured 24 h after incubation at 37°C 5%CO2. Levels of IL-2 (A) IFN-γ (B), TNF-α (C), IL-17A (D), IL-4 (E), IL-5 (F), IL-10 (G)and IL-13 (H) were determined by Cytokine Bead Array (BD Biosciences). No significant differences were seen cytokine levels produced in cultures restimulated with NaES. (TIFF) [file ppat.1002520.s002.tif]
