# Supplementary material for: Characterising the Mucosal and Systemic Immune Responses to Experimental Human Hookworm Infection
Source: PLoS Pathog. 2012 Feb 9;8(2):e1002520. doi: 10.1371/journal.ppat.1002520 (PMC3276555; doi:10.1371/journal.ppat.1002520)
Supplement: Table S1 — Sequences of primers used for real-time RT-PCR. (DOC) [file ppat.1002520.s003.doc]

| Protein name | Forward sequence (5'-3') | Reverse sequence(5'-3') |  |
| --- | --- | --- | --- |
| IL-2 | TGCAACTCCTGTCTTGCATT | TCAGTTCTGTGGCCTTCT TG |  |
| IL-4 | CTTTGTCAGCATTGCATCGT | CAGTTGTGTTCTTGGAGGCA |  |
| IL-5 | ACTCTGATGATAGCCAATGAGA | TCCAGTGTGCCTATTCCCTGA |  |
| IL-9 | AGGATGATCCACCGTCAAA | ATCAGTTGGGACGGAGAGAC |  |
| IL-10 | TGCCTTCAGCAGAGTGAAGA | GTCTTGGTTCTCAGCTTGGG |  |
| IL-13 | GTACTGTGCAGCCCTGGAAT | TTTACAAACTGGGCCACCTC |  |
| IL-15 | GGATTTACCGTGGCTTTGAG | ACATTCACCCAGTTGGCTTC |  |
| IL-17A | CCTCCAGAATGTGAAGGTCA | CTATCAGGGTCTTCATTGCG |  |
| IL-21 | AATCAAGCTCCCAAGGTCAA | TTTGGAAGGTGGTTTCCTC |  |
| IL-23 | GCTGTAATGCTGCTGTTGCT | GGATCCTTTGCAAGCAGAAC |  |
| IL-25 isoform 1 | CAGGTGTACAACCACTTGCC | TCCAGAAATGGGCAGAACTT |  |
| TGF-β1 | CACGTGGAGCTGTACCAGAA | GAACCCGTTGATGTCCACTT |  |
| IFN-γ | TGTTACTGCCAGGACCCATA | CTTCCTTGATGGTCTCCACA |  |
| Rorγt | CCAGTCCACTGATCTTGGGT | CAAGAGAGGTTCTGGGCAAG |  |
| GATA-3 | GGGCAATCAGTGTTACCGTT | ACCACCTTAGGCCAACTG AA |  |
| ALDH1A2 | AAGTTCCATTGTGCCAGGAC | TGGAAGAAGGGATGGAAGAA |  |
| FoxP3 | ACCACCTTAGGCCAACTGAA | CACAGATGAAGCCTTGGT CA |  |
| Tbet | GCTGCATATCGTTGAGGTGA | TGGTTGGGTAGGAGAGGAGA |  |
| IRF-4 | AGTTCAGCGGTTGAGGAGAA | CCTGAGAAATGGCACAGA CA |  |
| NKG2A | ATCAGCCCAGTGTGGATCTT | GAGCCCGACACAAATGCTA |  |
| NKG2D | CGAAAGTTACTGTGGCCCAT | GGTTGGGTGAGAGAATGGAG |  |
| β-actin | TGGAATCCTGTGGCATCCATGAAAC | TAAAACGCAGCTCAGTAACAGTCCG | |
